# How Can We Help Healthcare Workers during a Catastrophic Event Such as the COVID-19 Pandemic?

**DOI:** 10.3390/healthcare10061113

**Published:** 2022-06-15

**Authors:** Hannah Wozniak, Lamyae Benzakour, Christophe Larpin, Sebastian Sgardello, Grégory Moullec, Sandrine Corbaz, Pauline Roos, Laure Vieux, Typhaine M. Juvet, Jean-Claude Suard, Rafaël Weissbrodt, Jérôme Pugin, Jacques A. Pralong, Sara Cereghetti

**Affiliations:** 1 Intensive Care Unit, Geneva University Hospitals, 1205 Geneva, Switzerland; christophe.larpin@hcuge.ch (C.L.); jerome.pugin@hcuge.ch (J.P.); sara.cereghetti@hcuge.ch (S.C.); 2 Liaison Psychiatry and Crisis Intervention Service, Geneva University Hospitals, 1205 Geneva, Switzerland; lamyae.benzakour@hcuge.ch; 3 Department of Surgery, Geneva University Hospitals, 1205 Geneva, Switzerland; sgardello@outlook.com; 4 School of Public Health, University of Montréal, Montréal, QC H3T 1J4, Canada; gregory.moullec@umontreal.ca; 5 Occupational Health Service, Geneva University Hospitals, 1205 Geneva, Switzerland; sandrine.corbaz-kurth@hcuge.ch (S.C.); laure.vieux@hcuge.ch (L.V.); jean-claude.suard@hcuge.ch (J.-C.S.); jacques-andre.pralong@hcuge.ch (J.A.P.); 6 Haute École Arc Santé, HES-SO University of Applied Sciences and Arts Western Switzerland, 2000 Neuchâtel, Switzerland; pauline.roos@he-arc.ch (P.R.); typhainemaiko.juvet@he-arc.ch (T.M.J.); 7 School of Health Sciences, HES-SO Valais-Wallis, 1950 Sion, Switzerland; rafael.weissbrodt@hevs.ch; 8 Faculty of Medicine, University of Geneva, 1205 Geneva, Switzerland

**Keywords:** COVID-19, health promotion, health service, mental health, occupational health management, occupational stress, well-being, workplace

## Abstract

Healthcare workers (HCWs) have significantly suffered during the COVID-19 pandemic, reporting a high prevalence of anxiety, depression and post-traumatic stress disorder (PTSD). We investigated with this survey whether HCWs benefitted from supportive measures put in place by hospitals and how these measures were perceived. This cross-sectional survey, which was conducted during the first wave of COVID-19 at the Geneva University Hospitals, Switzerland, between May and July 2021, collected information on the use and perception of practical and mental health support measures provided by the hospital. In total, 3461 HCWs participated in the study. Regarding the practical support measures, 2896 (84%) participants found them useful, and 2650 (76%) used them. Regarding the mental health support measures, 3149 (90%) participants found useful to have the possibility of attending hypnosis sessions, 3163 (91%) to have a psychologist within hospital units, 3202 (93%) to have a medical nursing psychiatric permanence available seven days a week, and 3171 (92%) to have a hotline available seven days a week. In total, 436 (13%) HCWs used at least one of the available mental health support measures. During the COVID-19 pandemic, the support measures were valued by HCWs. Given the high prevalence of psychiatric issues among HCWs, these measures seem necessary and are likely to have alleviated the suffering of HCWs.

## 1. Introduction

Since March 2020, we have been facing a worldwide health crisis due to the COVID-19 pandemic. During the first wave of COVID-19 that in Switzerland started in March 2020, the Swiss government attempted to control the spread of the virus by imposing a semi-lockdown and by closing several supply chains such as shops, restaurants and recreational facilities. Hospitals had to reorganize themselves in order to adapt to the increased flow of patients. At the Geneva University Hospitals (HUG), which comprise eight public hospitals, the capacity of certain units was increased, working hours were modified from 8 to 12 h shifts, and the working environment was adapted to manage the infectious risk [1]. Furthermore, the HUG rapidly put in place several practical and mental health support measures to help healthcare workers (HCWs) during this crisis.

Practical support measures included complimentary food at the hospital, the delivery of groceries, free accommodation in hotels close to the hospital for staff living far away, as well as free parking.

Mental health support included (1) the availability seven days a week of a permanence with consultations by psychiatric physicians and clinical specialist nurses in psychiatry, (2) a psychological support service within hospital units, (3) a hotline available seven days a week, (4) hypnosis breaks performed by certified nurses and physicians. HCWs significantly suffered during this crisis, with two main categories of issues: (1) mental health issues; (2) risk of contamination by SARS-CoV-2. Regarding the first point, publications on the early impact on HCWs’ mental health showed a high prevalence of anxiety, depression and post-traumatic stress disorder (PTSD), with prevalence as high as 40%, 37% and 49%, respectively, in a recent metanalysis totalizing more than 53,000 HCWs [2,3,4,5,6,7,8,9]. Another study showed the prevalence of insomnia to be as high as 39% [10]. Interestingly, some authors found more mental health issues in intensive care unit (ICU) HCWs and in female HCWs, especially nurses [3,10]. Regarding other hospital workers such as administrative workers, they appeared to be scarcely represented in the current literature [2,3]. Concerning the second point which regards the risk of contamination by SARS-CoV-2, HCWs were faced with severely ill patients, with uncertainties about the risk of catching COVID-19 and transmitting it, as well as with the difficulties regarding life during lockdown [3,6]. Indeed, a recent metanalysis concluded that more than 50% of HCWs tested positive for SARS-CoV-2 during the pandemic [11]. It has been shown in previous studies that the fear of catching COVID-19 and passing it on to their relatives was a major source of stress for caregivers [3,6,12].

Many HCWs around the world expressed their discomfort during this period of crisis due to the difficult working conditions in overwhelmed healthcare systems [13]. Some individual strategies have been shown to benefit mental health in the context of the pandemic, including physical activity, maintaining social activities and sleeping enough [14,15]. Some authors have also suggested to promote these measures at the workplace [14]. In the emergency of the pandemic, several authors focused on producing guidelines on how hospitals can best support HCWs based on the experience of previous outbreaks such as severe acute respiratory syndrome (SARS) [16,17]. Recently, a study which focused on the support measures put in place for HCWs in Europe showed that the majority of countries had implemented support measures in various forms, ranging from psychological support to financial bonuses [18]. Other supportive workplace interventions have been described, such as the enhancement of communication between HCWs, the adaptation of the workplace structure (with, for example, the introduction of regular breaks and of relaxation areas, the adaptation of time tables), feedback sessions and training courses for HCWs to better deal with difficult situations [15,19]. However, the use and effectiveness of these support measures have not yet been studied.

Therefore, we decided to investigate whether the urgent supportive measures put in place to address potential mental health issues were used by HCWs and how these measures were perceived. Furthermore, we wished to describe whether ICU HCWs, among the most exposed staff to COVID-19, have other needs compared to non-ICU HCWs. This could be of help in the event of future outbreaks or healthcare crises to better address the needs of HCWs and to assist them in the most effective way.

## 2. Materials and Methods

### 2.1. Participants and Procedures

This descriptive cross-sectional study on the use and perception of the usefulness of extraordinary measures put in place by the HUG to support HCWs is part of a large longitudinal study on employee’s mental health and coping strategies during the COVID-19 pandemic, which has already lead to the publication of several studies [3,20]. In this study, all HUG employees, including clinicians such as physicians, nurses, care assistants, physiotherapists, ergotherapists, and non-clinicians, including administrative workers, domestic service staff and patient transporters, were contacted via professional email list servers with a request to participate in the study by answering an online questionnaire on the RedcapTM^®^ platform. Participation in the study was voluntary. Data were collected from 28 May to 7 July 2020, until the end of the first wave of COVID-19 in Switzerland, which ended in mid-May 2020.

Regarding practical support measures, which included free hospital meals, delivery of groceries to the hospital, free parking and hotel beds close to the hospital, participants were asked if they had used these measures and if they had found them useful.

Regarding mental health support measures, participants were asked if they had used the medical nursing psychiatric clinic, the psychological service, the seven-days-a-week hotline and finally if they had used the hypnosis service. In addition, they were asked if they found the availability of these supportive measures useful. They were also asked whether a psychological follow-up could be useful to them.

This study was approved by Geneva’s Regional Research Ethics Committee (BASEC ID 2020-00935).

### 2.2. Statistical Analyses

The analytical sample included HCWs who agreed to participate in the study. Socio-demographic data according to ICU status were collected. For our first objective concerning the use and the perceived usefulness of the availability of the provided measures, we carried out descriptive analyses according to ICU status. To assess whether there was a difference in needs between ICU and non-ICU HCWs, chi-square tests were performed. To further investigate whether working in the ICU was associated with the use of mental health support measures, we performed a multivariate logistic regression, adjusting the estimates for gender, age and profession. Two-tailed *p*-values at 0.05 were considered statistically significant. Due to the low number of missing data, a complete case analysis was applied. All analyses were performed using Stata^®^ IC 16.0 (StataCorp, College Station, TX, USA) and R Statistical Software (v1.3.1073; R Core Team 2021).

## 3. Results

### 3.1. Demographic Characteristics of the Sample Population

Of the 13,570 employees of the HUG invited to answer to the study questionnaire, 3461 (25%) accepted to participate. In the study population, 2561 (74%) participants were women (Table 1). Seven hundred and sixty-seven (22%) participants were single, and 2215 (64%) were married. With regards to their occupational category, 438 (13%) HCWs were physicians, 1341 (39%) were nurses, 261 (7%) were care assistants, and 1420 (41%) were other workers. Within the ICU, 352 HCWs participated in the study, which represents 69% of all the ICU HCWs (352/510).

### 3.2. Use of Practical and Mental Health Support Measures Implemented by the Hospital

The use of practical support measures is presented in Table 2. Regarding the practical support measures, 2650 (76%) individuals in the studied population used them. Regarding the mental health support measures, 436 (13%) HCWs used them. When comparing ICU to non-ICU HCWs, in total, ICU HCWs used more mental health support measures (27% vs. 11%).

### 3.3. Factors Associated with the Use of Mental Health Support Measures

After adjustment to gender, age and profession, working in the ICU was associated with the use of mental health support measures (Table 3). Being a woman and working as a physician, nurse or care assistant compared to other workers was also associated with the use of these measures.

### 3.4. Perceived Usefulness of the Measures Implemented by the Hospital

The perceived usefulness of the support measures is presented in Table 4. Considering the practical support measures, 2896 (84%) participants found them useful. Regarding the mental health support measures, 3149 (91%) HCWs found it useful to have the possibility to undergo hypnosis session, 3163 (91%) to have the support of a psychologist within hospital units, 3202 (93%) to have a psychiatric medical nursing permanence available seven days a week, and 3171 (92%) to have a hotline available seven days a week. Regarding the differences between ICU and non-ICU HCWs, we found that ICU HCWs were more prone to ask for a psychological follow-up than non-ICU HCWs (41% and 36%, respectively, *p* = 0.04).

## 4. Discussion

This study presents both the practical and the mental health support measures put in place by the HUG, which comprise eight public hospitals in Switzerland, during the first wave of the COVID-19 crisis. It highlights that, globally, the existence of these support measures was highly appreciated by HCWs, although the mental health support measures were less used compared to the practical support measures. Working in the ICU was highly associated with the use of mental health support measures.

There are few studies that focus on the support measures during the COVID-19 pandemic and, to our knowledge, no study has measured the rate of use of these measures or whether their existence has been perceived as useful by HCWs [19,21]. However, these measures are difficult to implement in the context of a crisis and require a lot of time and effort. Thus, it seems necessary to evaluate them in order to provide guidance in the event of a future crisis. Our study has another advantage in that it includes eight different professional categories among HCWs and is not limited to nurses (39%) and physicians (13%); thus, it provides a general representation of all hospital workers. This adds new information to the scarce literature regarding these other professional categories which have also been impacted by the COVID-19 pandemic and are at risk of mental health issues [2].

Considering the practical support measures, the HUG, like other hospitals, put in place several extraordinary measures to help HCWs [22]. These practical support measures were widely used (76%) and perceived as useful (84%). These measures might have improved the quality of the working conditions of HCWs and thus possibly helped to reduce their mental strain. As these practical measures are quite simple and fast to implement, they can be recommended in similar crises.

Regarding the mental health support measures, our study showed that HCWs found the existence of these services during the peak of the pandemic useful. Hypnosis breaks and support by a psychologist consulting directly within the hospital units were more used than the hotline and the psychiatric medical nursing permanence. An explanation could be that these services were offered directly in the unit and thus were easily accessible to HCWs. Thus, mental health support measures might be proposed directly in unit in future outbreaks.

Among all HCWs who responded to the survey, 13% used at least one of the mental health support measures offered by the HUG; this value increased to 27% when considering ICU HCWs. This rate appears very low in comparison with the answers concerning the perceived usefulness of the mental health support measures, which was close to 90%. We can first hypothesize that some HCWs were reluctant to use these services because they felt ashamed about their perceived failure to cope with this catastrophic situation without help. Furthermore, as some support services were present in the units, HCWs may have felt embarrassed to use them in front of their colleagues and might have preferred external support measures. Another hypothesis might be that HCWs did not have the time to use these support measures, due to the heavy workload during that time. Finally, it is possible that the mental health support measures were not used because of a delayed onset of mental health issues [6]. However, our study did not investigate the reasons for the use or non-use of these services, and therefore we cannot conclude on this point.

Even if few HCWs did use these services, the existence of these supports measures was perceived as useful by HCWs. We can argue that these mental health services for HCWs had a beneficial effect even if they were not used because it felt reassuring to know that these measures existed and that HCWs’ potential suffering was recognized. Although the effects of all these measures could not directly be assessed in our study due to its cross-sectional design, they may have limited the psychological impact of the crisis, which could have been worse without them. A study conducted during the SARS epidemic in 2003 in Singapore found that having support from colleagues and supervisors was associated with better mental health outcomes [23]. Given the high rates of anxiety, depression and the risk of PTSD found in various studies on the mental health of HCWs during the COVID-19 outbreak and the risk of delayed psychiatric disorders [3,4,5,6,12], the question arises as to whether longer-term mental health support could be beneficial. Mental health support and, especially, easy access to psychiatric consultations for HCWs should continue to be offered, even after the end of the COVID-19 pandemic.

Our study highlighted that ICU HCWs were more likely to use mental health support measures than non-ICU HCWs. Our previous study comparing mental health outcomes of ICU and non-ICU HCWs highlighted that ICU HCWs had a higher prevalence of anxiety, depression and low well-being [3]. During the COVID-19 crisis, ICU HCWs were constantly exposed to severely ill patients and witnessed numerous and unforeseen deaths [6]. This might explain the greater use of these supportive measures by ICU HCWs. Another explanation might be that mental health support measures were more readily available in the ICU rather than in other hospital units, which might have encouraged more ICU HCWs to use these services. Other factors associated with the use of these mental health support measures were being a female and working as a nurse, care assistant or physician. Previous studies found more mental health issues in female HCWs, especially nurses [4,10,24]. Although care assistants are less represented in previous studies, one study showed that these caregivers reported high peritraumatic distress compared with other ICU HCWs [3]. This could explain the increased use of these services by these categories. Therefore, particular attention should be given to these HCWs.

Some limitations in our study need to be acknowledged. Only 25% of the total HCWs responded to the study questionnaire, and a selection bias is therefore possible. However, within the ICU, 69% of all the ICU HCWs responded. This cross-sectional study only offers a description of the perceived usefulness and of the use of several support measures. Future longitudinal studies should focus on assessing whether these measures are having a positive impact on the short- and long-term mental health outcomes of HCWs. Studies focusing on the underlying process leading to the use or absence of use of these support services would also be helpful in the future. Lastly, we could only analyze the support measures that were implemented in our hospitals. Other support measures have also been described as useful in the literature, such as working on an effective and clear communication between HCWs, the adaptation of the workplace structure (with, for example, the implementation of regular breaks and of relaxation areas, the adaptation of time tables), training courses for HCWs to better deal with difficult situations and the promotion of good health behaviors such as providing access to physical activity facilities [14,15,19,25].

## 5. Conclusions

In conclusion, the present descriptive study highlights the use and perception of support measures implemented by a public hospital in the context of the COVID-19 pandemic and emphasizes that they were widely valued and used by HCWs and even more by ICU HCWs. Given the high prevalence of mental health issues among HCWs, these measures seem necessary and might have alleviated the suffering of HCWs. Particular attention should be given to female HCWs and to HCWs working in the ICU and in direct contact with patients. The time during which these support measures should be maintained is still discussed, since delayed mental health issues are to be expected. These measures could also be completed by other supportive measures, such as working on an effective communication with HCWs, the adaptation of the workplace structure, and offering training courses for HCWs to help them cope with difficult situations.

## Figures and Tables

**Table 1:** Descriptive characteristics of the participants (*n* = 3461).

	Total		ICU		Non-ICU	
**Overall**	3461	(100)	352	(10)	3109	(90)
**Sex**						
Women, *n* (%)	2561	(74)	234	(66)	2327	(75)
Men, *n* (%)	897	(26)	118	(34)	779	(25)
**Age**						
18–29 years old, *n* (%)	402	(12)	39	(11)	363	(12)
30–39 years old, *n* (%)	815	(24)	130	(37)	685	(22)
40–49 years old, *n* (%)	1032	(30)	97	(28)	935	(30)
50–59 years old, *n* (%)	1049	(30)	75	(21)	974	(31)
≥60 years old, *n* (%)	163	(4)	11	(3)	152	(5)
**Marital status**						
Single, *n* (%)	767	(22)	85	(24)	682	(22)
Married, *n* (%)	2215	(64)	242	(68)	1973	(63)
Divorced, *n* (%)	451	(13)	25	(8)	426	(14)
Widow(-er), *n* (%)	27	(1)	0	(0)	27	(1)
**Profession**						
Physician, *n*(%)	438	(13)	68	(19)	370	(12)
Nurse, *n* (%)	1341	(39)	198	(56)	1143	(37)
Care assistant, *n* (%)	261	(7)	32	(9)	229	(7)
Others, *n* (%)	1420	(41)	54	(16)	1366	(44)

Legend: Values are expressed in numbers and percentages.

**Table 2:** Use of practical and mental health support measures by HCWs.

	Total		ICU		Non-ICU		***p*** **Value**
**Overall**	3461	(100)	352	(10)	3109	(90)	
**Use of practical support measures**							0.054
Yes, *n* (%)	2650	(76)	255	(72)	2395	(77)	
No, *n* (%)	811	(24)	97	(28)	714	(23)	
**Use of any mental health support measures taken by the HUG**							<0.01 *
Yes, *n* (%)	436	(13)	94	(27)	342	(11)	
No, *n* (%)	3025	(87)	258	(73)	2767	(79)	
**Use of hypnosis breaks**							<0.01 *
Yes, *n* (%)	215	(6)	81	(23)	134	(4)	
No, *n* (%)	3246	(94)	271	(77)	2975	(96)	
**Use of the proximity psychologist service**							0.7
Yes, *n* (%)	185	(5)	17	(5)	168	(5)	
No, *n* (%)	3276	(95)	335	(95)	2941	(95)	
**Use of the psychiatric medical-nursing permanence**							0.11
Yes, *n* (%)	68	(2)	3	(1)	65	(2)	
No, *n* (%)	3393	(98)	349	(99)	3044	(98)	
**Use of the Hotline**							0.06
Yes, *n* (%)	105	(3)	5	(1)	100	(3)	
No, *n* (%)	3356	(97)	347	(99)	3009	(97)	

Legends: Values are expressed in numbers and percentages. * *p* < 0.05. Practical support measures defined as: free hospital meals, delivery of groceries to the hospital, free parking and hotel beds close to the hospital. Mental health support measures defined as: hypnosis breaks, proximity psychologist, psychiatric medical nursing permanence, hotline.

**Table 3:** Factors associated with the use of mental health support measures provided by the HUG.

*n* = 3461	Use of Mental Health Support Measures, Odds Ratio (95% CI)	*p* Value
**ICU workers**	2.6 (2–3.4)	<0.01 *
**Gender, female**	1.4 (1.1–1.8)	0.02 *
**Age**		
18–29 years old	Ref.	NA
30–39 years old	0.9 (0.7–1.4)	0.8
40–49 years old	0.9 (0.7–1.4)	0.9
50–59 years old	0.8 (0.6–1.2)	0.3
≥60 years old	0.9 (0.5–1.6)	0.6
**Profession**		
Physician	1.5 (1.1–2.2)	0.03 *
Nurse	2.2 (1.7–2.8)	<0.01 *
Care assistant	3.8 (2.7–5.4)	<0.01 *
Others	Ref.	NA

Legend: * *p* < 0.05.

**Table 4:** Perceived usefulness of practical and mental health support measures by HCWs.

	Total		ICU		Non–ICU		***p*** **Value**
**Overall**	3461	(100)	352	(10)	3109	(90)	
**Perceived usefulness of practical support measures**							<0.01 *
Useful, *n* (%)	2896	(84)	324	(92)	2572	(83)	
Useless, *n* (%)	565	(16)	28	(8)	537	(17)	
**Perceived usefulness of available hypnosis breaks**							0.1
Useful, *n* (%)	3149	(91)	328	(93)	2821	(91)	
Useless, *n* (%)	312	(9)	24	(7)	288	(9)	
**Percevied usefulness of having a proximity psychologist**							0.45
Useful, *n* (%)	3163	(91)	318	(90)	2845	(92)	
Useless, *n* (%)	298	(9)	34	(10)	264	(8)	
**Percevied usefulness of the psychiatric medical nursing permanence**							0.06
Useful, *n* (%)	3202	(93)	317	(90)	2885	(93)	
Useless, *n* (%)	259	(7)	35	(10)	224	(7)	
**Usefulness of the Hotline**							0.07
Useful, *n* (%)	3171	(92)	317	(90)	2854	(92)	
Useless, *n* (%)	290	(8)	35	(10)	255	(8)	
**Usefulness of a potential psychological follow-up**							0.04 *
Yes, *n*(%)	1271	(37)	145	(41)	1126	(36)	
Maybe, *n*(%)	1061	(31)	112	(32)	949	(31)	
No, *n*(%)	1129	(32)	95	(27)	1034	(33)	

Legends: Values are expressed in numbers and percentages. * *p* < 0.05. Practical support measures defined as: free hospital meals, delivery of groceries to the hospital, free parking and hotel beds close to the hospital. Mental health support measures defined as: hypnosis breaks, proximity psychologist, psychiatric medical-nursing permanence, hotline.

## Data Availability

Not applicable.
